# Nanotechnology in ligature-induced periodontitis: protective effect of a doxycycline gel with nanoparticules

**DOI:** 10.1590/S1678-77572010000400003

**Published:** 2010

**Authors:** Marco Antonio BOTELHO, Jose Galberto MARTINS, Ronaldo Sousa RUELA, Dinalva Brito QUEIROZ, Wagner Sousa RUELA

**Affiliations:** 1 Post Doctor in Orthodontics, Federal University of Ceará, Fortaleza, CE, Brazil.; 2 Doctor in Chemistry, Regional University of Cariri, Fortaleza, CE, Brazil.; 3 Doctor in Dentistry, Scholar of National Research and Development Council CNPq, FUNORTE School of Dentistry, Montes Claros, MG, Brazil.; 4 Scholar of National Research and Development Council CNPq, Fortaleza, CE, Brazil.

**Keywords:** Nanotechnology, Doxycycline, Periodontitis, Atomic force microscopy, Gel

## Abstract

**Objectives:**

The aim of this study was to test the efficacy of a locally applied 8.5% nanostructured doxycycline (DOX) gel in preventing alveolar bone loss in experimental periodontal disease (ePD) in rats by using the tapping mode atomic force microscopy (AFM).

**Material and Methods:**

ePD was induced in 24 Wistar rats. Animals were treated with the doxycycline gel topically, immediately after ePD induction, and 3 times a day during 11 days. Four groups (n=6) were formed as follows: Naïve group (animals not subjected to ePD nor treated); non-treated (NT) group (animals subjected to ePD, but not treated); vehicle gel (VG) group (animals subjected to ePD and treated with topical gel vehicle); and DOX group (test group): animals subjected to ePD and treated with the 8.5% DOX gel. In order to investigate topographical changes in histological sections, a novel simple method was used for sample preparation, by etching sections from paraffin-embedded specimens with xylol.

**Results:**

Comparing the AFM images, several grooves were observed on the surface of the alveolar bone and other periodontal structures in the NT and VG groups, with significantly greater depths when compared to the DOX group (p<0.05).

**Conclusions:**

Periodontal structures were brought into high relief confirming to be a simple and costeffective method for AFM imaging with ultrastructural resolution. The doxycycline gel was able to afford periodontal surface preservation, with flatter grooves.

## INTRODUCTION

Several methods have been employed in dentistry to better understand periodontal disease process^5^. The induction of periodontal disease by ligature placement is widely used in animal studies^1-4,7,8^. However, as criticism of this model still remains, other methods are necessary for a better understanding of the pathogenesis of periodontal disease.

Atomic force microscopy (AFM) is a very powerful tool as common technique for biological imaging, from molecule to cell morphology or even ultrastructural changes on tissue, where nanometric details are required to investigate the surface of the specimen. AFM is commonly used in examining biological specimens since it works well without any need for staining or coating^5,17^.

Periodontal structures, such as the periodontal ligament and alveolar bone, play an important role not only in maintaining structural integrity, but also in determining tissue function. Alveolar bone and attachment loss due to periodontal diseases, for example, is directly associated with the presence of dental biofilm and poor oral hygiene^4,6,9^.

In general lines, the pathogenesis of periodontitis involves the continuous presence of an irritating factor (e.g.: bacterial byproducts) that may initiate a local inflammatory reaction in predisposed hosts^7^. Insight into pathological structure function relationships depends on an accurate identification of ultrastructural changes on periodontal structures since the progression of periodontitis is considered a relevant cause of tooth loss in adults. Periodontitis is as a chronic inflammatory disease that affects a major part of world’s population characterized by localized or generalized bone resorption^8^.

Periodontal disease is also associated with the heart diseases. Different therapies are available to treat this pathology using nanotechnology for microencapsulating thymol, carvacrol and other natural flavonoids that produce antiinflammatory and antimicrobial effects during this inflammatory processes^8-10,21^. The host’s inflammatory response leads to edema, and the release of inflammatory mediators, causing periodontal pocket formation, loss of connective tissue attachment and alveolar bone resorption, ultimately leading to tooth loss^4,6,9^.

It is difficult to establish the periodontal status without periodontal tissue integrity conditions. The main difficulty seems to be greater when a three-dimensional evaluation is required^11,17^. The third dimension is important for the comprehensive understanding of tissue ultrastructure. The inclusion of topographical and roughness surface aspects might increase the prediction of periodontal therapy evaluation^22^.

As no experimental studies have investigated the possibility of using AFM to evaluate the integrity of periodontal components, clarification of the contributions of this technique on ultrastructural changes in the periodontal structures would be useful. The aim of this study was to test the efficacy of a locally applied 8.5% nanostructured doxycycline (DOX) gel in preventing alveolar bone loss in experimental periodontal disease (ePD) in rats by using a previously described tapping mode AFM technique^5^.

## MATERIALS AND METHODS

### Animals

Twenty-four male Wistar rats from the same nest (similar age; weight: 160-200 g) from our own animal facilities were housed in temperaturecontrolled rooms and received water and food *ad libitum*. The rats were randomly assigned to test and control groups using blocked randomization from a computer-generated list. All experiments were conducted in accordance with the local guidelines on the welfare of experimental animals and with the approval of the Committee of ethics in Animal Research of the Federal University of Ceará.

### Gel preparation

The doxycycline gel containing nanospheres was prepared at the Biotechnology Laboratory of evidence Pharmaceuticals, School of Pharmacy, Federal University of Ceará, Brazil, and kept in the dark to avoid any interference of light. For preparation of the 8.5% nanostructured doxycycline (w/w) (Merck Chemicals Ltd. Beeston, Nottingham, england) gel, Carbopol-94O (BF Goodrich Co., Cleveland, OH, USA) 2% (w/v) was used by mechanical dispersion in distilled water under vigorous agitation and polisorbate 80 was used, being neutralized until pH 6.0 with trietanolamine.

The nanostructured gel was prepared by the combination of two techniques as a Patent Application at National Institute of Industrial Property (INPI). The gel was stored in white polyethylene containers kept hermetically sealed under refrigeration at 8°C until used.

Stability study to evaluate the consistency of the gel over a period of 2 months was conducted by keeping the formulation at different conditions (4°C, 37°C and room temperature) and measuring the viscosity of the gel formulation at regular intervals. The viscosity was measured by a Brooke-field synchrolectric viscometer. The TD bar spindle of LV series was employed for the measurement. The study indicated that the viscosity of the carbopol gel did not change significantly throughout the stability period in the specified conditions.

### Induction of EPD

A sterile nylon (000) thread ligature (*Point Suture do Brasil Indústria de Fios Cirúrgicos Ltda*. Fortaleza,Ce-Brazil) was placed around the cervix of the second left upper molar of rats anesthetized with 10% chloral hydrate (400 mg/kg, i.p.), as described elsewhere^5^. The ligature was knotted on the buccal side of the tooth, resulting in subgingival position palatally and in supragingival position buccally. The contralateral right side was used as the unligated control animals were weighed daily.

### Drug Treatments

Four groups of 6 animals each were formed as follows: Naïve group (animals not subjected to ePD nor treated); non-treated (NT) group (animals subjected to ePD, but not treated); vehicle gel (VG) group (animals subjected to ePD and treated with topical gel vehicle); and DOX group (test group): animals subjected to ePD and treated with the 8.5% DOX gel (Dental Gel^®^; evidence Pharmaceuticals LTD., Fortaleza, Ce, Brazil). The topical treatment with the gel was performed applying 1 g of the product on the ligated sites immediately after the surgical procedure and 3 times a day, until the sacrifice at the 11^th^ day.

### Measurements of Alveolar Bone Loss

The animals were sacrificed on the 11^th^ day of periodontitis induction, and had their maxillae excised and fixed in 10% neutral formalin. Both maxillary halves were then defleshed and stained with 1% aqueous methylene blue in order to distinguish bone from teeth. The horizontal alveolar bone loss, the distance between the cusp tip and the alveolar bone, were measured using the method described by Botelho^5^ (2009).

To confirm these findings another digitalized technique was performed with the ImageJ (Research Services Branch, National Institute of Mental Health, Bethesda, Maryland, USA). A public domain Java image processing program were the area was determined not only in linear terms but also in square millimeters (mm^2^)as well. The measurement of alveolar bone loss was done by a blind examiner (M.A.B.) for both groups. The processing program calculated the area in pixels values, than statistics was applied in defined selections areas (distances and angles) in each periodontal area. The spatial calibration was performed in the photograph with a regular millimeter paper to provide real world dimensional measurements in units such as millimeters.

### Histopathological Analysis

After sacrifice by anesthetic overdose, animals had their maxillae excised. The specimens were fixed in 10% neutral buffered formalin and demineralized in 7% nitric acid. These specimens were then dehydrated, embedded in paraffin, and sectioned along the molars in a mesiodistal plane, for hematoxylin and eosin staining. Sixmillimeter-thick sections, which included the roots of the first and second molars, were utilized. The areas between the first and second molars, where the ligature was placed, were analyzed under light microscopy using a four-point (0-3) scoring system, considering the inflammatory cell influx, and alveolar bone and cementum integrity, as described previously^4,5^: Score "0" (zero): absence or only mild cellular infiltration (inflammatory cell infiltration is sparse and restricted to the region of the marginal gingiva), preserved alveolar process and cementum. Score 1: moderate cellular infiltration (inflammatory cells present all over the attached gingiva), little alveolar bone resorption and intact cementum. Score 2: intense cellular infiltration (inflammatory cells present in both gingival and periodontal ligament), accentuated degradation of the alveolar process, and partial destruction of cementum. Score 3: severe cellular infiltrate, complete resorption of the alveolar process, and severe destruction of cementum. The histopathological analysis was performed by a blind examiner (M.A.B.) in all groups.

### Histological sample preparation for AFM images

After routine histological sample preparation, chemical etching with 1% xylol was done, providing of semi thin sections from paraffin-embedded specimens to melt the upper layers, removing the superficial roughness caused by the edge of the microtome knife and bringing into high relief the biological structures hidden in the bulk. Fivemillimeter-thick sections, which included the roots of the first and second molars, were used. The areas between the first and second molars, where the ligature was placed, were analyzed using taping mode AFM. The samples were prepared and the aspects of its topography and ultrastructural changes were analyzed.

To obtain AFM height data, samples of rat maxilla were scanned in air with a Nanoscope IIIa Multimode AFM (Digital Instruments, Santa Barbara, CA, USA) by tapping mode at a scan of about 0.400 Hz, resonance frequencies of ca. 200 to 380 kHz, with crystal silicon cantilevers (Digital Instruments) at spring constant of approximately 40 N/m, and tip radius of 15 nm. The scan sizes performed were 30x30 µm. AFM scan controls were properly adjusted (sufficient contact force, and high gains) to avoid tip artifacts during the scanning of the samples. To 3D-visualization the height data were processed with Nanoscope software (Digital Instruments), version 5.12 r3. We used Nanoscope software to calculate the surface roughness for the periodontium regions AFM height data with scan size of 30x30 µm. Thirty regions per sample were randomly chosen to determine the mean surface roughness (*Ra*)^17,22^, and the topographical changes independent analysis were performed by a blind examiner (M.A.B.) for both tested groups.

### Statistical Analysis

The data are presented as the mean ± SeM or as the medians, where appropriate. A univariate analysis of variance (ANOVA) followed by Bonferroni’s test was used to compare means, and the Kruskal-Wallis test was used to compare medians. A probability value of *P* <0.05 was considered to indicate significant differences. A p*-value* <0.05 was considered significant. Analysis was performed with Graph Pad Prisma Version 3.0 software (GraphPad Software Inc. San Diego, CA, USA).

## RESULTS

### Effect of DOX gel on the alveolar bone loss

**Figure 1:**
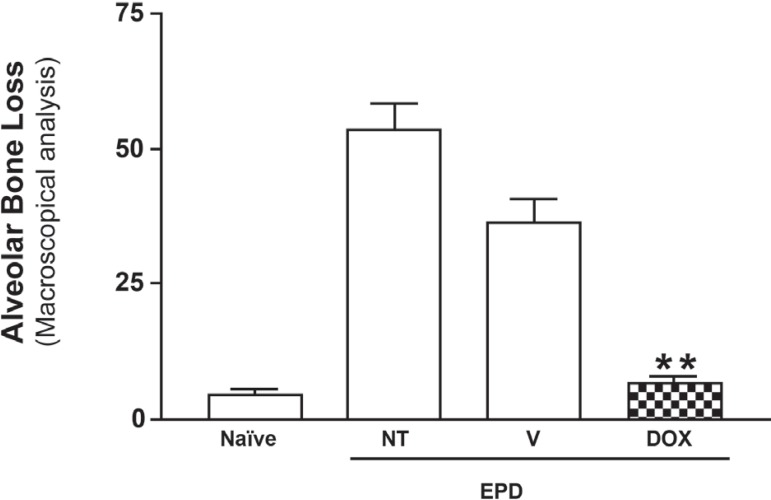
Macroscopic aspects of the effect of a nanostructured 8.5% Doxycycline (DOX) locally applied gel (Dental gel^®^) on alveolar bone loss. Experimental periodontal disease was also induced in the animals from the Non-Treated (NT) and in vehicle (V) groups. Naïve group was not submitted to EPD and received no treatment. Bars represent the mean ± Standard Error of Mean (SEM) of alveolar bone loss (mm; N=6). Asterisk indicates a statistically significant difference between the DOX group and NT group (*P<0.05; Bonferroni, ANOVA)

**Figure 2:**
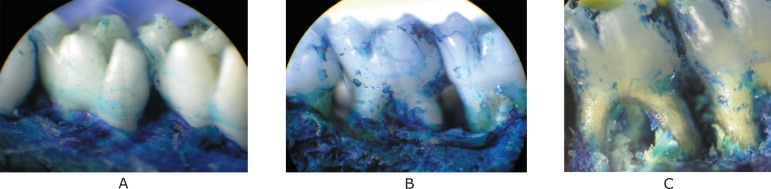
Macroscopic aspects of the effect of c on experimental periodontal disease in rats. A: macroscopic aspect of a normal periodontium of rat maxillae showing healthy periodontal structures; B and C: periodontium of rats subjected to induction of experimental periodontitis with no treatment (NT) and vehicle gel treatment, showing severe alveolar bone loss

Macroscopically, the treatment of animals subjected to 11 days of experimental periodontal disease with the DOX gel reduced the alveolar bone loss. These changes reached statistical significance (P<0.05), as compared to the untreated animals subjected to ePD and those treated with vehicle gel (Figure 1). These data can be clearly seen in Figure 2a, which shows the macroscopic aspects of the contra lateral right side (unligated side) with no resorption of the alveolar bone, compared to the severe bone resorption with root exposure in the untreated group (NT) and VG group (V) (Figure 2b and Figure 2c).

### Effect of DOX gel on the myeloperoxidase activity on rat gingiva

**Figure 3:**
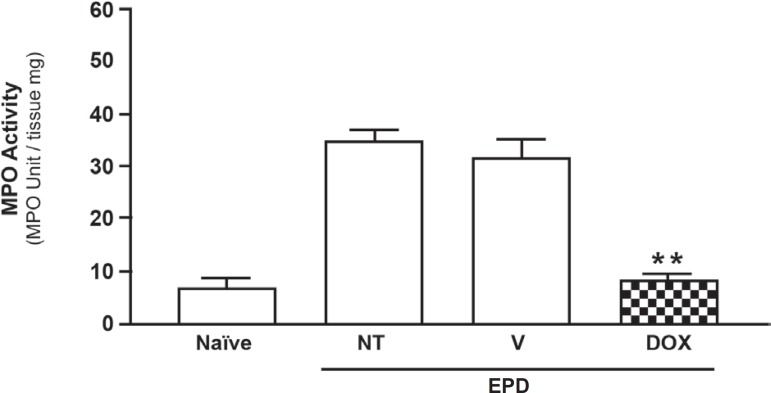
Effect of 8.5% DOX gel on myeloperoxidase (MPO) activity in the maxillary gingival tissue of rats submitted to experimental periodontal disease (EPD). Vehicle (V) gel and Doxycycline (DOX) gel were administered topically in animals subjected to EPD induction. EPD was induced also in Non-Treated (NT) animals and Naïve group received no treatment and was not submitted to EPD induction. Bars represent mean ± Standard Error of Mean (SEM) of the activity of MPO/mg of tissue. *P<0.05 was considered significantly different compared to NT group (ANOVA; Bonferroni’s test)

Figure 3 shows a reduction of inflammatory cell infiltration found in the periodontium tissue of animals subjected to experimental periodontitis and treated with the DOX gel. The neutrophil infiltration was evaluated by the myeloperoxidase activity in the gingival tissue. It was observed a significant (*P*<0.05) decrease in the myeloperoxidase activity in the gingival tissue only in the DOX group as compared to the rats treated with vehicle gel.

### Effect of DOX gel on the periodontal structures

**Figure 4:**
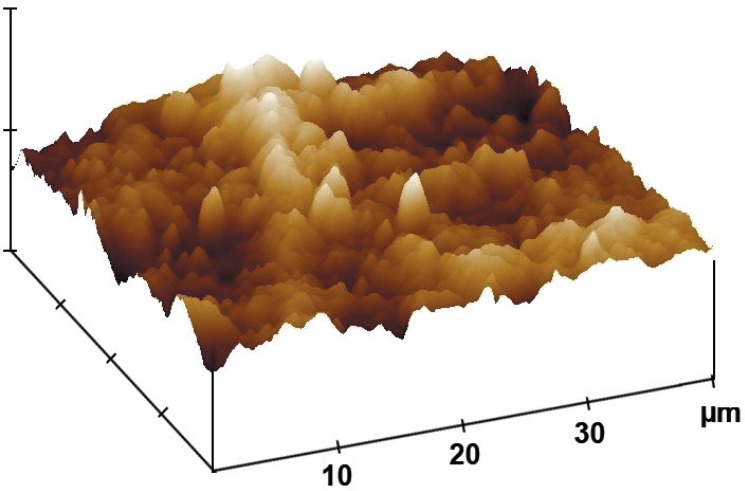
Effect of 8.5% Doxycycline (DOX) gel on alveolar bone surface roughness in rats subjected to experimental periodontal disease (EPD). The AFM image from the DOX group shows a regular bone surface covered with a shallow defined layer

**Figure 5:**
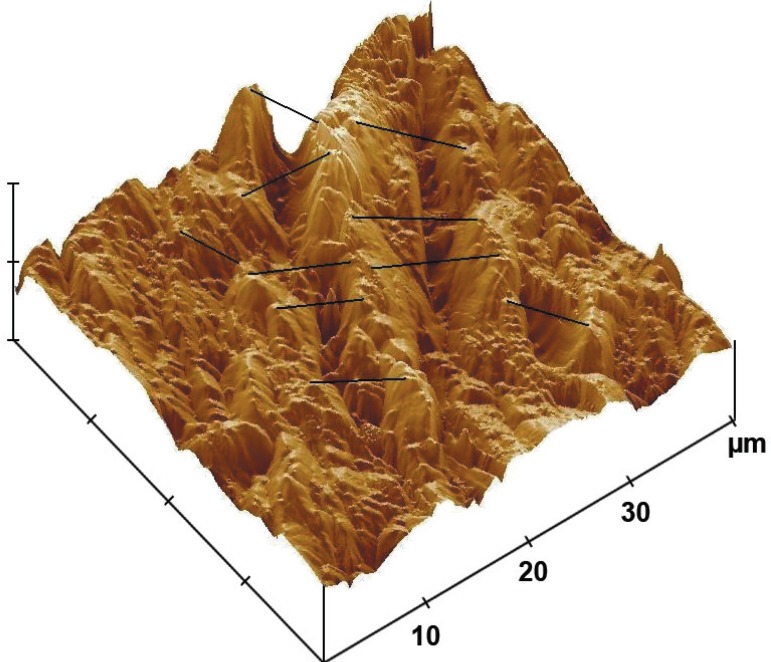
Chosen areas to evaluate the topographical changes in alveolar bone of rats subjected to the experimental periodontitis disease EPD treated with vehicle gel

The alveolar bone surface morphology of the DOX group was less altered compared with the vehicle gel group. The AFM image of the DOX group (Figure 4) shows a regular surface covered with a shallow defined layer. The height of this layer varied between 150 and 250 nm, while the NT group and the VG group varied between 750 and 950 nm (Figure 5).

**Figure 6:**
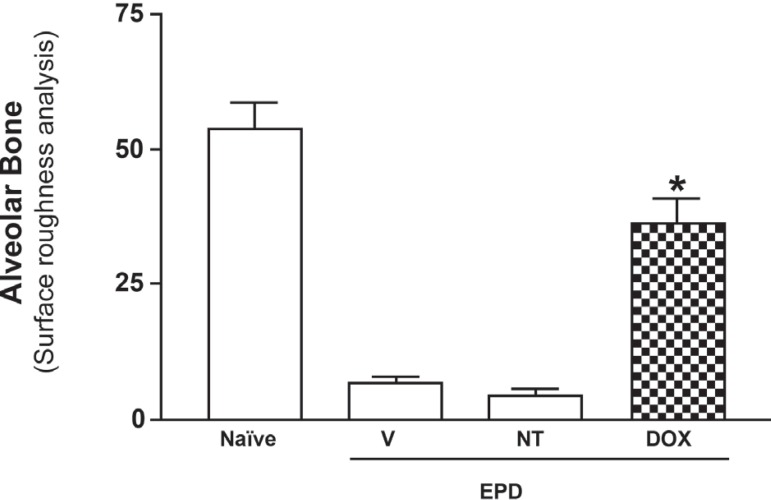
Effect of 8.5% Doxycycline (DOX) gel on the alveolar bone loss in alveolar bone roughness analysis by AFM in rats with experimental periodontal disease (EPD). The 8.5% DOX gel was administered topically, daily, in animals subjected to the EPD. Bars represent the mean ± Standard Error of Mean (SEM) of alveolar bone loss (mm; N=6). Asterisk indicates a statistically significant difference between treated group and Non-Treated (NT) group (*P<0.05; Bonferroni, ANOVA)

After 11 days of treatment with the DOX gel, the AFM images showed alterations compared with the V and NT group images (Figure 6). The depths of the grooves on the DOX group were 30-120 nm and their width ranged from 100 to 250 nm. After 11 days of periodontal challenge, the VG and NT groups presented grooves becoming more irregular than the DOX-treated grooves. These alterations were found in all of the six examined areas of all 6 specimens.

**Figure 7:**
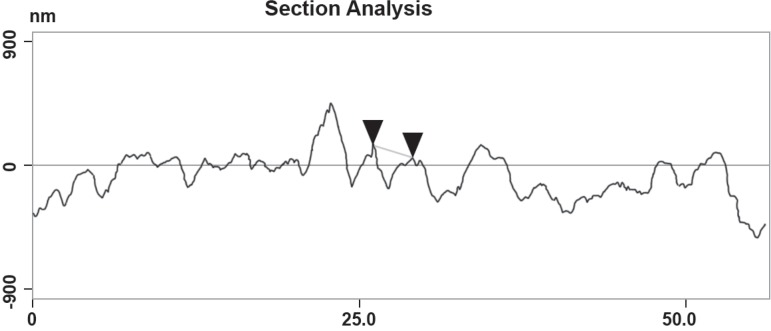
Topographical changes in alveolar bone surface of rats subjected to experimental periodontitis disease EPD and treated with 8.5% Doxycycline (DOX) gel

**Figure 8:**
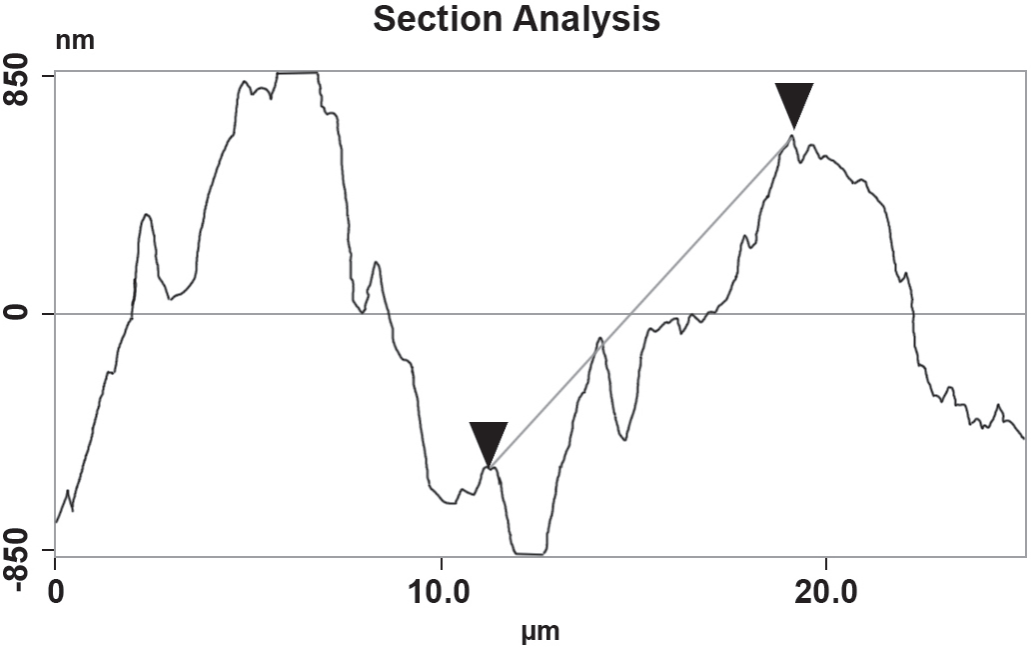
Topographical changes in alveolar bone surface of rats subjected to experimental periodontitis disease EPD treated with the vehicle gel

These data can be clearly seen in Figure 7, which shows the topographical aspects by section analysis of the alveolar bone of the rat maxillae with less resorption, and maintenance the integrity of the periodontal ligament, when compared to the severe topographical alterations caused in the NT group and vehicle gel treated group. Figure 8 shows the section analysis in bone surface on animals subjected to ePD and treated with the DOX gel.

### Effect of DOX gel on the periodontal ligament

The AFM analysis of the region between the first and second molars shows topographical alterations of the periodontium of the animals subjected to periodontitis that received VG group revealed a intense topographical alterations with deeper grooves and valleys on its structures surfaces with cementum destruction and complete alveolar process resorption (Figure 8), whereas a reduction of inflammatory cell infiltration and a partial preservation of the cementum and of the alveolar process was found in the periodontium of animals subjected to ePD and treated with the DOX gel, the deep grooves values were statistically significant (P<0.05), when compared to the NT and VG groups.

The AFM images of periodontium treated with the DOX gel showed morphological differences compared to the AFM images of the NT and VG groups. Moreover, the AFM images of the cementum treated with DOX gel was also different from images of the NT group.

Figure 5 shows how the AFM surface roughness analysis was made to establish the width of alveolar bone grooves after 11 days of treatment with the vehicle gel and the DOX gel. The surface of the Naïve and DOX groups seems relatively regular, with higher coefficients of roughness surface, in other hand, vehicle (V) and EPD groups presented greater alveolar bone resorption with much less roughness at the evaluated sites (Figure 6). Comparing the AFM surface roughness analysis, it can be stated that the periodontal structures in the DOX and Naïve groups seemed rougher when compared to the NT and VG groups. The mean depth of the groove in the DOX group varied between 90 and 350 nm and the width ranged from 1.0 to 1.5 μm. As the same results were obtained in all of the five examined areas for each specimen, the morphology in DOX group represents the typical surface morphology seen in the cementum treated with 8.5% w/w DOX gel.

## DISCUSSION

The local application of the DOX gel evaluated in this study played a positive role on the bone loss associated to experimental periodontal disease. This effect was associated with reduction of the inflammatory reaction. Since the histopathological and AFM analysis were done by a blind examiner for the test and control groups, the results of the present study demonstrated that locally applied controlled-release DOX gel might partly counteract the negative effect of periodontal plaque byproducts on periodontal healing.

The DOX gel showed an intense antiinflammatory activity inhibiting neutrophil infiltration as early as 6 h after ePD induction, as seen by the reduction of MPO activity on the gingival tissue. This effect was associated with a reduction in the local inflammatory cell infiltration. Perhaps this effect was achieved because the gel has a potent antibacterial activity^5^. Tetracycline and its derivatives have been shown to be inhibitors of matrix metalloproteinase that are part of the inflammatory response and contribute to the tissue breakdown in the periodontal disease^5^.

**Figure 9:**
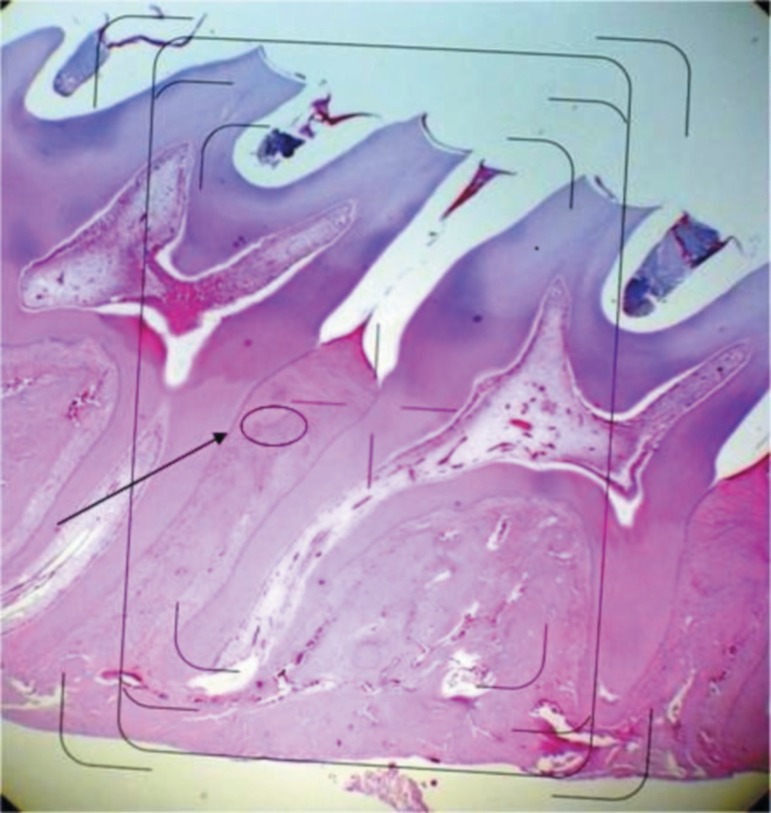
Histopathology from the periodontium of rats subjected to periodontitis. 8.5% Doxycycline (DOX) gel was administered topically immediately after ligature placement and daily after periodontitis induction for 11 days. Photomicrograph of the region between the first and second molars of rats: A represents the region evaluated in all groups. Hematoxylin-Eosin (HE) staining

Topical application of active substances offers an additional option in periodontal therapy^5,7,8^. In this present study, a nanostructured gel was able to protect the periodontal structures in this model. This effect was probably achieved by a controlled released system provided by nanospheres loaded with DOX, as we can see the preservation of alveolar bone in Figure 9. DOX gel was studied in smokers to investigate its inhibitory effect on experimental periodontal disease, showing expressive results on this particular group of patients^20^ as in other treatment groups^12^.

Previous studies have shown that DOX is one of the most potent and cost-effective TTC commercially available^13,14,16^, this clinical approach has been shown to reduce collagenase production by osteoblasts and osteoclasts and also to delay osteoclast recruitment following dental surgery^15^. DOX, as other chemically modified tetracyclines (CMT), also possesses other pharmacologic properties that may positively contribute for periodontal healing process^3,18,19^. Previous *in vitro* studies have demonstrated that DOX inhibits proteases by blocking the conversion of latent proteases into active mature forms and the activation of MMP’s by chelating metal ions^23,24^. In a rat model, CMT reduced the activity of tissue degradation enzymes such as myeloperoxidase and down-regulated bone resorption^5^. Also, systemic administration of low doses of DOX in humans, which have negligible antimicrobial effects, resulted in reduction of collagenase in gingival crevicular fluid^24^. Hence, since an increased protease activity is associated with the induced periodontitis, these properties of DOX may offer an additional explanation to the observed improved treatment outcome in this rat model that received this locally delivered DOX system compared to a vehicle gel only.

As shown in the alveolar bone height image in Figure 4, the use of the DOX gel for 1 min three times a day shows integrity of the alveolar bone roughness surface, while in Figure 5 the AFM images show a partially destroyed alveolar bone surface height image.

Concerning the topographical and roughness analysis, Figure 6 shows the height integrity of alveolar bone in Naïve group, while in the vehicle gel group (Figure 7) is clear to observe the narrower width and shallow height on the DOX group as well. We noticed the strong contrast between the VG group when compared to the DOX group, which clearly showed the presence of alveolar bone integrity. For better understanding of the method, Figures 5 and 8 show a clear perspective on how the AFM regions were measured from a regular histopathological section etched with xylol and then from the AFM specimens the program could measure the surface roughness and topographical changes with high accuracy.

Based on the findings of this experiment, AFM methodology can be used to evaluate with a systematic precision the alveolar bone structure and its surface regularity. The sections in Figures 7 and 8 show the alveolar bone surface, with 670 nm in mean reaching statistical difference when compared to the DOX group versus vehicle gel group, approximately 50% of the surface shows a significant difference (p<0.05) on roughness and topographical analysis after 11 days treatment. These findings lead us for a clearly demonstration that the treatment of an 8.5 % w/w DOX nanostructured gel does prevents denaturation of periodontal structures, especially the alveolar bone loss in this model.

## CONCLUSION

In summary, this study demonstrates a novel method to determine periodontal integrity in experimentally induced periodontitis. The fact that tetracycline and its derivatives are currently used as chronic treatments for periodontal disorders with a safety profile highlights the need for further research in order to demonstrate their clinical use. It was also demonstrated that AFM is a powerful tool used to evaluate the phases of the process of experimental periodontal disease.
